# Danish GP trainees’ experiences of navigating patient expectations for non-indicated procedures or tests: a qualitative interview study

**DOI:** 10.1080/02813432.2025.2597784

**Published:** 2025-12-17

**Authors:** Anna Dines Hansen, John Brandt Brodersen, Alexandra Brandt Ryborg Jønsson

**Affiliations:** a Department of Public Health, University of Copenhagen, Copenhagen, Denmark; b Centre for Cancer and Organ Diseases, Copenhagen University Hospital, Rigshospitalet, Denmark; c Research Unit for General Practice, Department of Community Health, Arctic University of Norway, Tromsø, Norway; d Department of People and Technology, Roskilde University, Roskilde, Denmark

**Keywords:** Medical education, trainees, patient, patient demand, primary care, general practice

## Abstract

**Objective:**

To explore how trainee doctors in general practice navigate patient expectations and requests for non-indicated procedures or tests, and what factors may pressure them into practising defensively.

**Design and setting:**

A qualitative interview study with 13 GP trainees from the Capital Region and Region Zealand in Denmark. An interview guide was developed based on existing literature and a pilot interview with an experienced GP. Data were coded in NVivo (version 15) and analysed using thematic analysis.

**Results:**

The trainees described several challenges when facing patient requests for unnecessary procedures or tests. These challenges were linked to their own uncertainty, patient expectations and behavior, as well as external factors. The trainees described experiencing self-doubt and uncertainty, particularly when their clinical judgement was questioned by patients. Managing patient expectations, especially when patients were worried, sought tangible evidence, or had private health insurance, was described as challenging. Additionally, clinical values and a stressful environment was said to further influence decision-making processes. Together, these factors sometimes pressured the trainees into adopting a more defensive approach. With more experience, many developed greater confidence and argumentation, making it easier to refuse patient requests while maintaining a good relationship.

**Conclusion:**

Our study indicates that uncertainty, patient expectations, and external circumstances may, in certain situations, pressure Danish GP trainees into practising defensively. Managing and navigating both their own and the patient’s uncertainty emerges as a key challenge. Future research is needed to explore how trainees can be better supported and educated in managing uncertainty and patient expectations in general practice.

## Introduction

Over the past decades, there has been a growing interest in the individual maintenance of personal health. This includes a wide range of practices - from regular exercise and healthy eating to undergoing medical tests and examinations even in the absence of symptoms - all in the name of prevention. This has been described as part of a broader trend that elevates health to the status of a super value, a phenomenon often referred to as “healthism” [1]. Beyond individual pursuits of health, disease mongering, screening initiatives, and governmental emphasis on personal responsibility for prevention have also contributed to shaping public perceptions, sometimes fostering insecurity even among healthy individuals [2]. Along with easier access to health data, healthism and public campaigns may encourage apparently healthy individuals to request even more testing, such as additional scans and blood work [3]. While encouraging individuals to take responsibility for their health is often seen as positive, this growing emphasis on personal health can also have unintended consequences. One such downside is its role in driving overuse of healthcare resources and overdiagnosis, where people are unnecessarily labelled as patients despite the absence of meaningful symptoms or illness [4]. Yet, many individuals who initiate or request tests and examinations may not fully understand the potential consequences of these actions. Often, they are motivated by the widespread belief that in healthcare, “more is better” - the idea that more testing leads to better outcomes - without recognising the risks of false positives, unnecessary treatments, or the psychological harm associated with overdiagnosis [5]. This “more is better” logic is evident in general health checks, which have been shown to provide no clear benefit and, in some cases, to cause more harm than good, explaining why such practices are generally discouraged [6]. However, the pressure and demand for tests and procedures is increasingly present in primary care consultations and is sometimes described as contributing to the practice of defensive medicine - where doctors find themselves acting against their clinical judgment [7].

Defensive medicine has been defined in a legal and economic context as occurring when doctors order tests, procedures or visits, or avoid high-risk patients or procedures, primarily (but not necessarily solely) to reduce their exposure to malpractice liability [8]. However, Baungaard et al. in their systematic review of defensive medicine in European literature, found that over half of the studies adopted a broader definition of the phenomenon; one that encompassed not only concerns about malpractice liability, but also a range of other motives, including fear of patient dissatisfaction, fear of missing a serious diagnosis, and fear of negative publicity [9]. An example, where they use a broader understanding is a study among Danish general practitioners (GPs), who describe defensive medicine as “unnecessary and meaningless medical actions, carried out mainly because of external demands that run counter to the GP’s professionalism” [7]. Within this broader context, defensive medicine can be seen more as a dynamic and evolving process rather than a fixed, predetermined phenomenon, as it has often been portrayed in the literature on health economics and law [10].

Defensive medicine is closely related to the phenomenon of medical overuse, encompassing unnecessary or non-indicated procedures and tests. Similar practices are also described in the OECD report Tackling Wasteful Spending on Health under the concept of ‘low-value medical tests’ [11]. Here, we use the terms ‘unnecessary testing’ and ‘non-indicated procedures’ to denote such practices. A study of German GPs identified several drivers of medical overuse such as limited clinical experience and underdeveloped communication skills. The authors suggest that inadequate medical training may lead to diagnostic uncertainty and to further excessive testing as a perceived safeguard, especially among early-career doctors [12]. However, although Alber et al. acknowledge in their introduction that diagnostic uncertainty is a core premise of general practice - due to an unselected patient population and a low prevalence of manifest disease - they do not revisit this point in their discussion. Recognising this fundamental uncertainty is crucial, as it helps explain how factors such as limited clinical experience and communication challenges increase an already-present uncertainty, rather than create a new one.

Following this, this paper qualitatively explores how GP trainees experience and navigate situations where apparently healthy patients expect and request additional, non-indicated procedures or tests and what factors may pressure them into practising with a defensive approach.

## Methods

### Setting

This study was conducted in Denmark, a welfare state of around 5.99 million people [13]. The welfare system is mainly financed through taxes and ensures everyone has access to free public healthcare without co-pay [14]. All Danish citizens are assigned a default GP. The GPs are gatekeepers to the disease-specialised part of healthcare. Most Danish GPs have 15-minute consultations, conducting patient-centred consultations including point-of-care (blood)tests if clinically indicated. At times, they also act as intermediaries for insurance companies, as patients with private health insurance often ask their GP to provide a referral to access faster or additional treatment from private providers. In such cases, the GP’s task is to verify that there is a medical issue requiring further assessment or care before the insurance will cover private services [15]. Doctors in Denmark must complete at least 6 years of training following medical school to be authorised as a GP. This includes 1 year of internship followed by 5 years as a GP trainee, structured into 3 phases [16]: Phase 1 begins with 6 months in a general practice (referred to as the “base practice”) and continues with 24–30 months in various hospital departments, including monthly return days in the base practice. Phase 2 consists of 6 months in the base practice, while Phase 3 involves 12 months in a different general practice (not the base practice). In parallel with their clinical training, trainees also participate in a structured course program called SPEAM, which consists of training modules essential for becoming a GP [17].

### Recruitment and characteristics

GP trainees were recruited from the Capital Region and Region Zealand through social media posts and face-to-face recruitment at postgraduate courses, where ADH, a female medical student, presented the project. Participants were required to have at least nine months of clinical experience in general practice and be in the first or second phase of their specialisation in general practice. The final sample consisted of 13 trainee doctors (8 women, 5 men), aged 28 to 42 years. Eleven participants were in the first phase of their training, while two were in the second phase. Efforts were made to ensure diversity in gender, age, and practice location (rural or urban), and no participants withdrew (See Tables 1 and 2 for participant characteristics).

**Table 1.** Participants characteristics.

Characteristics	Number	Percentage (%)
Total	13	
Gender		
*Female*	8	61,5 %
*Male*	5	38,5 %
Age		
*25–30*	2	15,4 %
*31–35*	6	46,2 %
*36–40*	4	30,8 %
*41–45*	1	7,7 %
Practice type		
*Group (two or more)*	12	92,3 %
*Solo*	1	7,7 %
Practice location / Region		
*Capital Region*	9	69,2 %
*Region Zealand*	4	30,8 %
*Urban*	10	76,9 %
*Rural*	3	23,1 %

**Table 2.** Participants characteristics.

Characteristics		
Interview person	**Gender**	**Age**
I 1	F	28
I 2	F	29
I 3	F	35
I 4	F	31
I 5	M	42
I 6	F	31
I 7	M	33
I 8	F	32
I 9	F	35
I 10	M	38
I 11	M	37
I 12	M	38
I 13	F	36

### Data collection

The interview guide was developed based on a literature review and a pilot interview with one ex-perienced GP (see Appendix A). ADH conducted the semistructured interviews between October 22, 2024, and February 3, 2025. Interviews took place in various locations chosen by participants, includ-ing private homes, university offices, a café, and online settings. No prior relationship existed between ADH and the participants. The interviews began with informed consent and an introduction to ADH’s background and role as a medical student. This was followed by a brief explanation of defensive medicine and an introduction to the broader definition to ensure a shared understanding of the concept [7]. This clarification was intended to align participants’ interpretation of the term before the interviews proceeded. No other individuals were present during the interviews. The duration of interviews ranged from 40 to 80 min, and no repeat interviews were conducted. All interviews were audio recorded and transcribed verbatim. The audio recordings were deleted after transcription, and all contact in-formation was removed to ensure confidentiality and data security. ADH started transcribing and analysing after the first interview. This preliminary analysis aimed to change the interview guide and to ask new open questions. Interviews, notes, and transcriptions were produced in Danish. Only the extracts presented in this article were ad hoc translated into English.

### Data analysis

The study was designed as an exploratory investigation, allowing for flexibility during the interviews. While an interview guide was used to ensure consistency, participants were encouraged to bring up additional topics they found relevant. This approach resulted in an abductive methodological frame-work, where theoretical concepts informed the analysis, yet the process remained open to emerging themes and new insights from the empirical material. Following this, all data were coded using the NVivo software (version 15) and analysed using thematic analysis [18], guided by AJ, an experienced anthropologist and qualitative researcher. Clinical perspectives were assessed by JB, an experienced GP and professor of evidence-based medicine. In this process, the researchers read through the data to identify patterns of meaning and organised them into themes. The continuous analytic process, with a description of coding themes and a coding framework, was presented to and discussed with the other members of the author group at analytical meetings. After coding, different theoretical perspectives were explored, and the final interpretation represents what the research group agreed best illuminated the data. Data collection and analysis were guided by the concept of information power, ensuring that the sample held sufficient richness and relevance for the study aim.

We acknowledge that our prior research on overdiagnosis and the potential harms associated with defensive medicine may have introduced some preconceptions. At the same time, having a medical student as interviewer facilitated open discussions, as both she and the trainees could reflect and relate to each other’s experiences. Data is reported using the COREQ checklist [19].

## Results

We identified three major themes that trainees find challenging in their consultations with apparently healthy people requesting procedures or tests with no clinical indication:

1. Uncertainty and Personal Traits
2. Patient Expectations and Behaviour
3. External Circumstances

### Uncertainty and personal traits

#### Beginner’s self-doubt and uncertainty

During the interviews, trainees frequently reflect on their initial struggles with professional uncertainty, due to their ‘beginner’ status, describing how their developing clinical judgment makes it challenging to communicate clearly with patients about what should and should not be done. In some cases, even when they feel clinically confident in their plan, they describe difficulties gaining the patient’s trust, particularly with their body language and argumentation. As a result, some report practising defensively or with extra caution to ensure safety and to reassure patients who might question their competence. One trainee (I 10) highlights the difficulty of being a young trainee and showing authority to an older patient:

“It was difficult in the beginning [saying no]. Also because you had to be rhetorically skilled when you started as a young doctor, facing an older patient who looks at you and thinks: ‘Where are the adults?’” (I 10)

Another trainee (I 6) expresses the fear of making a confident decision with limited clinical experience, worrying that the decision may later be proven wrong:

“Well, so what happens to me is that, to some extent, I can feel professionally uncertain because I’m not that experienced. I mean what if I confidently insist that I believe this is the right approach, and then it actually turns out to be wrong?” (I 6)

The same trainee describes starting with a confident plan, but the patient’s uncertainty and persistency quickly undermines the trainee’s confidence, leading to a change in the plan:

“I might try the plan that I think myself. If they are persistent enough, and I don’t feel 100% convinced or sure, then sometimes I might end up going along with it.” (I 6)

Across the interviews, several trainees point to the concern of overlooking something serious as a central aspect of their uncertainty. Most of them describe this concern as more significant than the risk of facing a lawsuit or receiving a complaint, which some trainees acknowledge as an eventual reality in the medical profession. As one trainee (I3) puts it:

“The worst thing I can imagine is receiving a complaint and realising that I made a mistake that had consequences for the patient. I know I will get complaints eventually - we all do - but what truly bothers me would be if the complaint is justified, especially if it involves a serious error and harms the patient.” (I 3)

However, as their experience increases, many trainees describe a growing ability to stand by their clinical judgments. With more patient encounters, they develop stronger reasoning skills and begin to trust their pattern recognition and clinical judgement:

“And I think it [rejecting patient’s requests] was more difficult back then, because you hadn’t seen that many patients, right? I mean, now I’ve seen several thousand patients over the past few years. But at that time, I had not seen that many, so my evidence base wasn’t very large. But as I’ve gotten better, I also start to trust myself more.”(I 7)

On the other hand, one trainee (I 4) argues that not showing uncertainty and acting with excessive confidence could, in some situations, potentially be harmful, stating:

“I also don’t think a doctor should have too much confidence. So, if a patient comes in and says something, sometimes you just have to say: ‘Well… Maybe you’re right.’… Because I just think that if a doctor becomes too confident, then honestly, they might become a bit dangerous.” (I 4)

#### Personality and conflict avoidance

The personal dimension of the consultation, including aspects of the trainees’ personalities, plays a crucial role in clinical decision-making for some. Two trainees (I6, I8) describe themselves as conflict-averse or “people-pleasers,” making it difficult for them to refuse a patient’s request. They find themselves caught in a dilemma, and sometimes the desire to avoid a confrontation may lead to a more defensive approach, as one of them describes:

“There’s also a personal aspect to it. I avoid conflicts and don’t find them comfortable. I want the patients to like me (…). And if that professional uncertainty arises in me, I can sometimes be tempted to just go along with what the patient asks for. Because then, at least, I still have their approval, that they like me.” (I 6)

### Patient expectations and behaviour

A common theme across the interviews is the pressure trainees feel from patient expectations, particularly in navigating what several describe as a “consumer-like mindset”. One trainee (I 1) illustrates this by telling the consultation from the patient’s point of view as a shopping experience:

“It’s almost as if patients see the consultation like a store, where they can simply pick whatever they want from the shelves.” (I 1)

In addition to this consumer-like mindset, some trainees report situations where other actors, such as private health insurance companies, increase the patient expectations and place additional pressure on the trainees. A few trainees describe such situations where patients do not seek their medical judgement, but instead use the doctor more as a gateway to services:

“I also think there are more demands now that many people have health insurances. I mean, they come and they’re not really interested in my judgement. They just want a referral: ‘Well, my health insurance covers - just give me a referral.’” (I 2)

The same trainee (I 2) emphasises the difficulty of convincing patients without offering something tangible in the consultation. If the consultation concludes without a visible or measurable outcome, patients may not feel entirely secure:

“It is the patient’s expectations that are the hardest to manage. Not in the sense that the patient demands and expects things in only a negative way, but because the patient cannot be fully convinced and does not feel entirely secure without having been scanned or measured. (…) It can be difficult to convince them that there is nothing wrong unless there is something that they see as objective.” (I 2)

To meet the patient’s expectations, some trainees describe feeling pressured to “give something” during the consultation, even if it means acting defensively and ordering unnecessary testing or referrals to maintain a good doctor-patient relationship. One trainee (I8) refers to this as part of “the show” and relates it to “the principle of the least restrictive means”:

“So with the ‘tired ones,’ I often take a broad panel of blood test including D-vitamin, thyroid function, haemoglobin, blood count, red and white blood cells, and so on (…) I mostly think that this won’t show anything. But again, in a way, I also feel that it’s part of the show… Well, I wouldn’t be able to say: ‘It’s just because you’re a bit depressed during winter.’ I mean, when I haven’t checked for a physiological cause. And sometimes blood tests are, I feel, a ‘the principle of the least restrictive means’.” (I 8)

Similarly, another trainee (I 1) reflects on using blood tests as a compromise, not necessarily because they are clinically relevant, but to meet the patient halfway:

“It definitely plays a role in making the argumentation easier, and the patient feel that something has happened. It can also be a bit of a compromise: ‘We have taken blood tests, so maybe I haven’t referred you to a scan, but the blood tests were fine.’ So it becomes a compromise with the patient. We’ve done something - just not exactly what you had in mind. It’s not like we haven’t done anything at all.” (I 1)

Other trainees express a more dismissive approach. They report that they would only swab if a potential treatment is available, but not in cases where it is likely a viral infection. This illustrates a balancing act performed by many of the trainees - on the one hand, they weigh whether a test is un-necessary, and on the other, they prioritise maintaining a good relationship with the patient:

“Many patients want to be tested for COVID and influenza, and many doctors do it. However, for me, if there is no potential treatment, I choose not to do it.” (I 9)

Several trainees distinguish between “the worried ones” and “the demanding ones”. While both patient groups may express strong expectations, the underlying motives may differ. “The worried ones” often present a concern with more diffuse and vague symptoms, which can evoke further un-certainty in the trainee. In contrast, “the demanding patients”, who make highly unrealistic requests, can sometimes, but not always, be easier to push back against, as one trainee (I 6) describes:

“Yes, if it’s completely absurd, it’s easier. Then I can just say, ‘We’re not doing that.’ But if it’s in that grey area, and I’m not completely sure about it professionally, I might bend a little.” (I 6

However, another trainee (I 9) describes some requests as reflecting an underlying concern that should be approached with caution, as giving in can trigger what the trainee refers to as “an avalanche”:

“Sometimes it comes out as a demand, but it’s really an expression of an underlying concern. But I think that’s where you must be really careful not to give in, because then it just becomes an avalanche rolling. There’s always something new that can be investigated.” (I 9)

While managing patient expectations can be challenging, several trainees agree that providing well-reasoned explanations often reassures patients, leading them to accept the clinical decision. As one trainee (I13) points out:

“People want an explanation, and if they get it, they are generally satisfied. If you don’t explain it to them, they’re not satisfied. But that makes a lot of sense. Otherwise, they feel deceived in some way.” (I 13)

In addition, some trainees describe writing unusually detailed or lengthy notes in the medical record, particularly when they decide not to refer or pursue further investigations. As a strategy, some also mention discussing the plan with their supervisor to ensure clinical safety and to share responsibility. Discussing the plan, composing such notes, and providing well-grounded explanations often takes extra time and preparation – resources that are not always available to a trainee doctor in a busy clinical setting.

### External circumstances

#### Clinical values

Most of the trainees have worked in different clinical settings throughout their careers and have ex-perienced various values in their workplaces. Some have experienced clinics with a more restrictive approach to unnecessary testing, while one trainee (I 2) describes a former workplace with half of the consultations more like secretarial work than “real doctor” work:

“Yes, it was quite a dull everyday routine. During half of the consultation, you could have just hired a secretary.” (I 2)

A few trainees describe how they occasionally go along with more defensive practices than they personally believe are necessary. For instance, one trainee (I 2) shares how the tutor doctors in her clinic use CT scans more frequently than best available evidence suggests:

“It’s more that the threshold for ordering CT scans is quite low in my practice, which sometimes puts pressure on me. Or ‘pressure’ is maybe the wrong word, I don’t really mind it, so I simply go along with it. However, there are many CT scans I wouldn’t have referred if I had been in my KBU general practice. There is a bit of a difference.” (I 2)

Another trainee (I 11) describes the tutors in the clinic as having a more relaxed approach to unnecessary testing, prioritising patient satisfaction and avoiding potential conflicts:

“I have some tutors who are rather relaxed about it [ordering unnecessary tests], and would rather avoid complaints, preferring a happy patient than an upset one.” (I 11)

#### Time and preparation

In addition to differences in clinic culture, the structure of general practice and the time available per consultation may also play a crucial role in clinical decision-making. Some trainees describe time pressure as a contributing factor in giving in to patient requests, particularly when they are behind schedule and have limited time to prepare for the consultation. One trainee (I 7) reflects on how it often requires additional effort to explain the reasoning behind refusing a patient’s request, and under time pressure, saying “yes” can feel like the quickest and easiest way:

“It’s about dealing with patients and spending time on that. Often, it’s just easier to say, ‘Fine, you’re right,’ so I can move on and get through my 15-minute time slot, rather than sitting there trying to explain something to a layperson.” (I 7)

Similarly, another trainee (I 8) emphasises how even a small amount of preparation time can impact the consultation outcome:

“That’s what I mean when I say that if I have some time to reflect and see what kind of patient I’m about to meet, then I’m a bit better prepared. Compared to when I’m completely behind and just call in the next patient without even looking at the schedule, then I’m not prepared at all. So definitely, in those cases, I’m much more likely to just be like: ‘Next.’”

Finally, one trainee (I 11) points out that calm communication can significantly improve the consultation and the relationship, even if it means spending just 30 s longer:

“We work under pressure, and time is always a factor. The next patient is practically knocking on the door, so we want to move quickly. But if you take just 30 seconds to two minutes to calmly explain things, you build trust and reach a shared understanding of the plan.” (I 11)

## Discussion

### Principal findings

The 13 interviews indicate that Danish GP trainees, particularly early in their careers, experience challenges when apparently healthy patients request or expect non-indicated tests or procedures. These situations can evoke feelings of self-doubt and uncertainty, especially when trainees struggle to fully convince the patients or their clinical decisions are questioned. Managing patient expectations, especially when the patients seek tangible evidence, are worried or hold private health insurance, can be challenging for the trainees. Navigating these challenges often requires well-founded argumentation, compromises during the consultation, support from supervisors, or detailed documentation of decisions. Additionally, clinical values and a stressful clinical environment with limited preparation time may further influence clinical decision-making. The trainees describe that, with more experience, many develop greater confidence and stronger argumentation skills. However, taken together, uncertainty, patient expectations, time and clinical values, may sometimes pressure the trainees into adopting a more defensive approach, potentially leading to overuse of medical resources and undermining the role of gatekeeping in primary care.

### Findings in relation to other studies

Our findings on uncertainty in clinical decision-making align closely with previous research. Managing uncertainty and the fear of missing serious conditions is a well-known challenge affecting doctors throughout their careers [20], and this seems to be particularly pronounced for the trainees who are still building clinical experience and confidence. Han et al. propose a detailed framework for understanding uncertainty in health care. They present a three-dimensional taxonomy based on its sources, issues, and locus to help navigate the complexities of uncertainty in medical practice [21]. In the context of a primary care setting, Kuehlein et al. illustrate how GPs encounter patients with non-specific symptoms and a low prevalence of manifest disease, contributing to more diagnostic uncertainty than in secondary care [22]. Thus, the types of un-certainty described in other studies are not only reflected in our findings but appear to be a defining aspect of being a trainee doctor.

Patient expectations for testing without clinical indication have also been explored in previous research. A population-based cross-sectional study from Portugal shows that the majority of adults believe medical tests should be performed almost annually - expectations that deviate significantly from evidence-based recommendations [23,24]. This highlights the considerable pressure GPs face, as well as the phenomenon of “healthism”, which refers to the idea that individuals are increasingly expected to take responsibility for their own health [1]. This resonates with our findings of patients’ consumer-like mindset. Martins et al. note the complexity that arises when the doctor and patient enter a consultation with fundamentally different understandings of what is medically necessary [24]. Similarly, trainees in our study describe how patients often expect to receive “something” from the consultation, regardless of the clinical indication. Some patients even hold private health insurance, which in certain situations adds additional pressure on the trainees, as also reported by GPs in both Norwegian and Danish studies [25,26].

Navigating these expectations and maintaining a good relationship was particularly challenging for trainees. Similarly, Walderhaug et al. found that Norwegian GPs used strategies to avoid unnecessary imaging, like the strategies reported by our trainees, such as consulting a professional authority – as the trainees do with supervisors – and offering alternatives to requested imaging, which we term “compromises” [27]. Both the Norwegian GPs and our trainees emphasized the importance of clear, reasoned arguments when rejecting requests. The Norwegian GPs also noted that factors such as a long-term doctor–patient relationship and trust seemed to influence both communication and how both parties experienced the decision [27]. Taking this into account, maintaining a good relationship with the patient can be even more difficult for trainees, as this trust has not yet been built. Brandsæter et al. have also investigated low-value imaging in Norway, and identified multiple drivers present at organisational, cultural, and individual levels [28]. This underscores that individual strategies used by the trainees and the experienced GPs to navigate patient expectations are embedded within broader systemic and cultural contexts, and efforts to reduce unnecessary testing should not only focus on individual decision-making but also consider healthcare system structures and work-related culture.

Together, these factors, including clinical values and time pressure, may contribute to a more defensive approach among the trainees. But giving in to the pressure can have unintended consequences. For example, a study by Adamu et al. found that prescribing antibiotics for acute respiratory infections in primary care probably modestly increases future reattendance for similar conditions [29]. In other words, giving in to pressure may reinforce unrealistic expectations and make future consultations even more difficult, which one trainee in our study referred to as starting “an avalanche.”

Our study does not quantify the extent of defensiveness, as some previous studies have done, making direct comparisons between trainees and more experienced GPs difficult. Still, the trainees indicate that they are more uncertain and have a more defensive behaviour earlier in their careers, suggesting a gradual shift as confidence and clinical experience increase. This aligns with findings from a scoping review on defensive medicine, which conclude that especially less experienced doctors are more likely to have a higher probability of a defensive pattern [30].

Baungaard et al. explored the phenomenon of defensive medicine in a European context and propose a broader definition, which resonates with the types of behaviour our trainees describe [9]. Most of our trainees do not describe ordering tests or investigations defensively primarily out of fear of litigation, although some report documenting more thoroughly when rejecting a patient’s request. Instead, most of their defensive behaviour arises when they try to meet patients’ expectations and struggle to manage both their own and the patients’ uncertainty. This perspective contrasts with Bester’s view on defensive medicine, who argues that responding to uncertainty with testing and investigations is not necessarily defensive medicine, as long as it is not driven by self-protection - an essential criterion in his definition [31]. Yet, our study presents a more nuanced perspective, where the trainees describe uncertainty - both their own and their patients’ - as a central driver of their defensive behaviour. This challenges Bester’s clear-cut distinction and underscores the complex, context-dependent nature of defensive medicine, which still lacks a universally agreed-upon definition and varies across clinical settings.

Equally important is the observation that the opposite of uncertainty - overconfidence - can also impair clinical judgment. As Berner et al. argue, this overconfidence can adversely affect decision-making and increase the likelihood of diagnostic errors [32]. One trainee in our study even reflects on the potential danger of becoming too confident, noting that it could result in “becoming dangerous.” This illustrates the delicate balance between confidence and uncertainty tolerance in clinical practice.

Altogether, these findings underscore the importance of addressing patient expectations early and setting clear boundaries around clinical decision-making. As several trainees point out, offering a well-grounded explanation and taking even 30 extra seconds more to communicate clearly can play a significant role in preventing unnecessary and defensive practices, even when patients are persistent. Patient-centred communication and shared decision-making are known strategies which can help address fears and expectations while aligning care with patient values [33]. These strategies may help reduce unnecessary interventions, but they rely on both doctor and patient acknowledging that uncertainty is an inherent part of medicine and that more is not always better.

### Strengths and limitations of the study

The semi-structured interview effectively captures the trainees’ personal experiences, thoughts, and emotions. To our knowledge, this is the first study to specifically explore how trainees experience patient expectations for investigations without a clear clinical indication, making it a valuable contribution to the field. Their perspective is particularly important, as they are close enough to the start of their careers to vividly recall medical school and their early clinical experiences, while also having worked across various healthcare settings. This combination allows for both reflective insight and a degree of breadth in their responses.

However, some trainees may have developed unconscious habits that this study does not fully capture, and what one trainee perceives as defensive behaviour may vary depending on the individual’s perspective and the clinical context. Additionally, those who chose to participate may have held stronger views or more pronounced experiences with demanding patients and defensive behaviour than those who did not, potentially influencing the findings. Nevertheless, we consider the data to hold sufficient information power to meaningfully describe the trainees’ experiences. Interpreting the findings in the context of this specific group provides a valuable contribution to understanding their perspectives and what for them are the most challenging

Finally, as our interview guide and analysis were framed around the concept of defensive medicine, some aspects may have been overlooked or not fully captured because our focus was directed elsewhere. A broader approach with more open-ended questioning in future studies may allow us to explore this more thoroughly and capture such nuances.

## Conclusion

This study highlights how uncertainty, limited experience, patient expectations, and external factors may, in certain situations, pressure Danish GP trainees into practising defensively. Managing and navigating both their own and the patient’s uncertainty emerges as a key challenge. Future research is needed to explore how trainees can be better supported and educated in managing uncertainty and patient expectations in clinical practice - skills that may help reduce defensive practices and prevent unnecessarily turning people into patients.

## Appendix A. Interview guide

**Table:**

Themes	Examples of questions
*Formalities, anonymisation consent, introduction to ADH and defensive medicine*	
Informant’s information	Age, gender, phase/time in clinical training, type of practice, practice location
Experiences and perceptions, patients’ requests and defensive behaviour	Do you often encounter situations where patients request tests, treatments, or referrals that you consider unnecessary? Have you ever felt pressured to act in a way you would describe as defensive in your clinical practice?
Giving in to requests	*If yes* Can you recall any examples or experiences with that? What led you to give in in that situation? How does it feel to go against your professional judgement? And what impact does it have on you?
Circumstances	Are there any circumstances that make you more likely to practice with a defensive approach? *If yes* What kind of circumstances? Who might be more challenging to say no to? *If the trainee does not mention it, ask in-depth about:* The patient’s presentation The trainee’s fear of missing something Fear of complaints or litigation Personal values Workload and time pressure
Going against the patient’s requests	Have you ever had experiences where you stood your ground with a patient insisting on a test or procedure? *If yes* What is it like to say no to a patient who insists? How does it feel? What impact does it have on you as a trainee?
Education	Do you feel that your training in medical school has adequately prepared you to handle patients who request unnecessary diagnostics or tests? Are you aware of how these patients affect you? -What aspects of your education could be improved in an ideal world to help you manage these patients better?
Conclusion	Do you have any questions? How was this for you? Is it okay if I contact you again if new questions arise?
